# Auditory Brainstem Representation of the Voice Pitch Contours in the Resolved and Unresolved Components of Mandarin Tones

**DOI:** 10.3389/fnins.2018.00820

**Published:** 2018-11-16

**Authors:** Fei Peng, Colette M. McKay, Darren Mao, Wensheng Hou, Hamish Innes-Brown

**Affiliations:** ^1^Key Laboratory of Biorheological Science and Technology, Chongqing University, Ministry of Education, Chongqing, China; ^2^The Bionics Institute of Australia, East Melbourne, VIC, Australia; ^3^Medical Bionics Department, University of Melbourne, Melbourne, VIC, Australia; ^4^Collaborative Innovation Center for Brain Science, Chongqing University, Chongqing, China; ^5^Department of Biomedical Engineering, University of Melbourne, Melbourne, VIC, Australia; ^6^Chongqing Engineering Research Center of Medical Electronics Technology, Chongqing University, Chongqing, China

**Keywords:** frequency-following response, voice pitch, resolved harmonics, unresolved harmonics, autocorrelogram, phase-locking value

## Abstract

Accurate perception of voice pitch plays a vital role in speech understanding, especially for tonal languages such as Mandarin. Lexical tones are primarily distinguished by the fundamental frequency (F0) contour of the acoustic waveform. It has been shown that the auditory system could extract the F0 from the resolved and unresolved harmonics, and the tone identification performance of resolved harmonics was better than unresolved harmonics. To evaluate the neural response to the resolved and unresolved components of Mandarin tones in quiet and in speech-shaped noise, we recorded the frequency-following response. In this study, four types of stimuli were used: speech with either only-resolved harmonics or only-unresolved harmonics, both in quiet and in speech-shaped noise. Frequency-following responses (FFRs) were recorded to alternating-polarity stimuli and were added or subtracted to enhance the neural response to the envelope (FFR_ENV_) or fine structure (FFR_TFS_), respectively. The neural representation of the F0 strength reflected by the FFR_ENV_ was evaluated by the peak autocorrelation value in the temporal domain and the peak phase-locking value (PLV) at F0 in the spectral domain. Both evaluation methods showed that the FFR_ENV_ F0 strength in quiet was significantly stronger than in noise for speech including unresolved harmonics, but not for speech including resolved harmonics. The neural representation of the temporal fine structure reflected by the FFR_TFS_ was assessed by the PLV at the harmonic near to F1 (4th of F0). The PLV at harmonic near to F1 (4th of F0) of FFR_TFS_ to resolved harmonics was significantly larger than to unresolved harmonics. Spearman's correlation showed that the FFR_ENV_ F0 strength to unresolved harmonics was correlated with tone identification performance in noise (0 dB SNR). These results showed that the FFR_ENV_ F0 strength to speech sounds with resolved harmonics was not affected by noise. In contrast, the response to speech sounds with unresolved harmonics, which were significantly smaller in noise compared to quiet. Our results suggest that coding resolved harmonics was more important than coding envelope for tone identification performance in noise.

## Introduction

Mandarin is a popular tonal language and has four lexical tones: flat tone, rising tone, falling then rising tone, and falling tone. The different tones in Mandarin help to convey the semantic information of speech sounds. For example, the syllable /ma/ with the four lexical tones “mā,” “má,” “mǎ,” and “mà” means “mother,” “fiber,” “horse,” and “scold,” respectively (Chao, 1965). The accurate perception of Mandarin tones plays a crucial role in communication and social life. The four lexical tones are primarily determined by the fundamental frequency (F0) contour (Howie, 1976).

The auditory system extracts the F0 contour from the harmonic structure of the sound (which varies over time in tonal speech sounds) (Plack et al., 2006). In the cochlea, the basilar membrane functions similarly to a bank of band-pass filters, and the bandwidth of the auditory filter increases with center frequency (Glasberg and Moore, 1990). However, the space between harmonics of a complex tone is constant. The resolvability of harmonics in this work was defined according to the theoretically derived properties of the bandwidth of the auditory filter (Glasberg and Moore, 1990): The low-order harmonics of a complex tone can be separated out by a single auditory filter and are called resolved harmonics. The resolved harmonics evoke distinct patterns of excitation on the basilar membrane. Several high-order harmonics (greater than approximately the 10th harmonic) (Bernstein and Oxenham, 2003) are represented together in a single auditory filter and are called unresolved harmonics (Plomp, 1967). The unresolved harmonics evoke a complex temporal pattern of activation on the basilar membrane whose envelope repeats at the fundamental period of the waveform. The auditory nerve fibers respond to the different patterns of basilar membrane activation evoked by stimuli with resolved and unresolved harmonics. For stimuli with only unresolved harmonics, the activation pattern in the auditory nerve thus corresponds to the envelope of the signal. For stimuli with only unresolved harmonics, the temporal discharge patterns in auditory nerve fibers will also reflect amplitude modulations (beating, interference patterns) produced through the interactions of nearby harmonics.

The F0 contour extracted from the resolved and unresolved harmonics have different contributions to Mandarin tone identification. Most studies have shown that tone identification accuracy for sounds containing the low resolved harmonics is nearly perfect (Stagray et al., 1992; Luo and Fu, 2006; Liu et al., 2014). Likewise, the envelope of speech can be used in quiet conditions to identify the tone, with about 80% correct identification relying on envelope cues alone (Whalen and Xu, 1992; Fu et al., 1998; Fu and Zeng, 2000). Tone identification with only envelope cues is worse than for when the low resolved harmonics are available. Studies also suggested that pitch perception is the basis of tone identification (Xu and Pfingst, 2003). The neural temporal representation of pitch has been studied in auditory nerve fibers in animal models (Cariani and Delgutte, 1996a, b), simulation models (Meddis and Hewitt, 1991; Meddis and O'mard, 1997;), and using the scalp-recorded frequency-following response (FFR) in humans (Gockel et al., 2011; Krishnan and Plack, 2011). The temporal coding theory of pitch is based on the idea that the auditory nerve fibers fire on average at a specific phase of the waveform (phase locking) (Moore, 2012). Neurophysiological studies (Cariani and Delgutte, 1996a,b) and temporal auditory nerve simulation models (Meddis and Hewitt, 1991; Meddis and O'mard, 1997;) have shown that auditory nerve fibers do indeed phase lock to the frequency of basilar membrane vibration at the place related to *each individual* resolved harmonic, and phase lock to the *envelope* of the excitation pattern of the basilar membrane for unresolved harmonics. Neurophysiological studies and models based on spike timing (Cariani and Delgutte, 1996a,b; Meddis and O'mard, 1997;) also predict that harmonic complexes consisting only of high (unresolved) harmonics should produce weaker F0 pitches than their counterparts consisting of low, resolved harmonics. The temporal coding theory based on spike timing also supports the findings from psychophysical studies in humans (Houtsma and Smurzynski, 1990) showing that the pitch strength of resolved harmonics is stronger than for unresolved harmonics of complex tones.

The FFR is thought to reflect phase-locked neural activity that is synchronized across whole populations of neurons in the rostral auditory brainstem (Worden and Marsh, 1968; Marsh et al., 1975; Smith et al., 1975; Glaser et al., 1976; Galbraith, 1994), i.e., it is the mutually-synchronized component of the brainstem response. Recent studies showed that the cortical response also contributed to the FFR revealed by MEG and fMRI (Coffey et al., 2016b, 2017b) and the subcortical structures dominate the neural response in the electrically recorded FFR (Bidelman, 2018). FFR has been used to explore the relationship between subcortical neural activity and speech perception in humans. The term “FFR” originally referred to the neural representation of the spectral information and the envelope of the stimulus, and was generated by fixed-polarity stimuli (Aiken and Picton, 2008). A number of studies have demonstrated that FFR can reflect the neural representation of the formants of speech (Krishnan, 2002; Russo et al., 2004; Aiken and Picton, 2006, 2008), the steady-state periodicity pitch in complex tones (Greenberg et al., 1978, 1987; Smith et al., 1978), and the time-varying voice pitch contours in speech (Krishnan et al., 2004, 2005; Dajani et al., 2005). The FFR autocorrelation magnitude is positively correlated with behavioral estimates of pitch salience using pure tones (Marmel et al., 2013), complex tones with different harmonic number (Krishnan and Plack, 2011) and non-speech noise (Krishnan et al., 2010a, 2012). For complex tones, Krishnan and Plack (2011) showed that the magnitude of the autocorrelation function of the FFR in response to resolved harmonics (4–10th of F0) was higher than unresolved harmonics (12–18th of F0).

When alternating-polarity stimuli are used, the neural response to each stimulus polarity can be either added to derive the envelope-following response (FFR_ENV_), or subtracted to derive the spectral-following response (FFR_TFS_). Adding the responses to positive and negative polarity stimuli has the effect of enhancing the representation of phase-locked neural activity to the *envelope* of the stimulus, and cancels the cochlear microphonic and electrical stimulus artifacts present in the recording that change with the polarity (Greenberg et al., 1987; Aiken and Picton, 2008). Compared to FFR_ENV_, FFR_TFS_ contains an enhanced representation of the temporal fine structure information. Compared to the results of Krishnan and Plack (2011) above, however, Zhu et al. (2013) showed that the phase-locking value (PLV) at F0 of envelope-following responses (FFR_ENV_) in response to complex tones with resolved harmonics (1–5th of F0) was lower than the response to unresolved harmonics (6–10th of F0). The FFR strength extracted from the autocorrelation function in the study of Krishnan and Plack (2011) combined the neural response to the envelope and fine structures of stimuli, and mathematically it is analogous to the magnitude of frequency-domain spectrum. However, the PLV measure used in the study of Zhu et al. (2013) measures the consistency of the phases of spectral components across trials. Zhu et al. (2013) also showed that there was no difference in the PLV at F0 of FFR_ENV_ between responses to broadband complex tones and sounds containing only unresolved harmonics. The authors suggested that FFR_ENV_ to broadband complex tones was mostly contributed by the neural response to the unresolved harmonics.

For vowels, Jeng et al. (2011) reported that peak autocorrelation magnitude and spectral F0 amplitude of FFR in response to the Chinese syllable /yi/ (with rising tone) did not change when the stimulus was high-pass filtered up to the 8th harmonic. The study of Jeng et al. (2011) suggests that the strength of F0 contour reflected by the FFR to the speech was not decreased when the resolved harmonics were filtered from the stimuli. Further studies have attempted to determine the relative contribution of the resolved or unresolved harmonics of speech to the FFR_ENV_ F0 strength. The first experiment in the study of Laroche et al. (2013) showed that the spectral F0 amplitude of FFR_ENV_ to stimuli including only the first formant (F1—dominated by the resolved harmonics) of the vowel /a/ with fixed F0 was similar to the stimuli only including the second formant (F2—dominated by the unresolved harmonics) in quiet. However, the stimuli only including F1 and F2 in the study of Laroche et al. (2013) both included resolved and unresolved harmonics. To our knowledge, it is not clear whether there is a difference in the strength of F0 contour reflected by the FFR_ENV_ to the resolved and unresolved harmonics of speech in quiet.

Psychological studies have shown that the neural mechanisms of extracting F0 from the resolved harmonics of complex tones are more robust than from the unresolved harmonics (Gockel et al., 2006). Gockel et al. (2006) also suggested that temporal phase-locking contributed to the precise coding of F0 for the resolved harmonics. Kong and Zeng (2006) also showed that tone identification for synthetic stimuli including only F0 and its harmonics was nearly perfect with a background of white noise (0 dB SNR). However, participants scored only 60% correct for stimuli containing only the envelope of speech with background noise at the same SNR. Another study suggests that the tone identification dominated by the auditory system response to the resolved harmonics in the noise condition (Liu et al., 2014).

It is of interest to explore whether the subcortical neural representation of speech could reflect the noise tolerance to resolved and unresolved harmonics of speech. The second experiment in the study of Laroche et al. (2013) showed that the spectral F0 amplitude of FFR_ENV_ to stimuli only including F1 was significantly greater than to stimuli only including F2 or F3 in white noise, and they suggested that the response at F0 of FFR_ENV_ to stimuli only including F1 (dominated by resolved harmonics) was more noise robust than stimuli only including F2 or F3 (dominated by unresolved harmonics). However, the masking effect of white noise for F2 or F3 would be stronger than for F1. It is not clear whether the FFR_ENV_ would reflect noise tolerance when the noise masking is balanced for the resolved and unresolved harmonics using filtered speech-shaped noise. Laroche et al. (2013) also showed that the FFR_TFS_ at F1 (the 7th harmonic) in noise was significantly lower than in quiet.

The aim of this study was to evaluate neural temporal information reflected by the FFR in response to resolved and unresolved harmonics of speech with a rising F0 curve in quiet and in speech-shaped noise. We recorded FFR to a single vowel with a rising tone from Mandarin speech, filtered to contain either all resolved or all unresolved harmonics, and presented the sound either in quiet or in noise.

## Materials and methods

### Participants

Eighteen adults (10 males, 8 females; ages 20–28 years old) were recruited for this study, and all of them participated in both the psychophysical and physiological experiments. All participants were native Mandarin speakers, and all showed normal hearing sensitivity with hearing thresholds lower than 15 dB hearing level (HL) at octave frequencies from 250 to 8,000 Hz. Each participant signed an informed consent in accordance with experimental protocols approved by the Human Research and Ethics Committee of the Royal Victorian Eye and Ear Hospital.

### Behavioral test—tone identification task

To determine whether the addition of noise affected tone identification differently when the stimuli contained either only resolved or only unresolved harmonics of the Mandarin speech, participants performed a tone identification task with an adaptively varying noise level.

Nine Chinese monosyllables (/ba/, /da/, /ma/, /ke/, /xi/, /du/, /yi/, /a/, /wu/) with four lexical tones (36 monosyllables in total) were recorded by a male speaker in a sound-attenuating booth. The signal was digitized at 44.1 kHz. The F0s of all speech ranged from 80 Hz to 180 Hz. The F0—curves of all speech estimated and extracted from the short-term spectrogram of speech by finding the maximum spectral energy in each time window (80 ms shifted in 2 ms). The duration of the speech stimuli ranged from 300 to 450 ms. Two versions (“resolved” and “unresolved”) of each stimulus were constructed by low-pass and high-pass filtering the signal, respectively. The cut-off frequency of the filter depended on the harmonic number (Bernstein and Oxenham, 2003), with stimulus frequencies greater than the 10th harmonic considered to be unresolved. Based on the F0 (80–180 Hz) of all speech, the cut-off frequency of low-pass filter and high-pass filter was 800 Hz and 2,000 Hz, respectively, which was constant for all speech. A low-pass filtered “resolved” version (“resolved stimuli”) was constructed for each speech stimulus by using a zero-phase 16th order Butterworth low-pass filter with the 3 dB cut-off at 800 Hz. Similarly, “unresolved” versions of each stimulus (“unresolved stimuli”) were made, using 16th order Butterworth high-pass filter with cut-off 2,000 Hz.

Speech-shaped noise was used to control the SNR in noise conditions. Speech-shaped noise was generated from the speech samples in a multi-step process. First, an averaged speech signal was generated by averaging all the speech stimuli. Second, the spectrum of the averaged speech signal was calculated by Fourier transform, and then the phase of the averaged speech signal was randomized. Third, the inverse Fourier transform was conducted to generate the speech-shaped noise. Two versions of noise (“low-frequency” and “high-frequency”) were generated using the same filters that were used for constructing the resolved and unresolved speech in noise conditions, respectively. The frequency spectra of the noise were the same as the speech spectrum.

For the adaptive procedure in noise conditions, playback of the noise started 300 ms before the speech stimulus began and continued until 300 ms after the speech stimulus finished. The speech signals were normalized to the same root-mean-square level and were calibrated using the sound level meter (Norsonic 140) to 65 dBA (Fmax).

In order to mask distortion products for unresolved stimuli, a low intensity (45 dBA) filtered pink noise was used. The pink noise was low-pass filtered in Audacity (Audacity, 2017) with a cutoff at 1,500 Hz (48 dB/octave) and played back continuously for unresolved stimuli in both quiet and noise conditions.

To find the lowest SNR at which participants could perform the tone identification task with 79.4% correct identification, an adaptive one interval four-alternative forced-choice (4AFC) task was used. Four buttons with lexical tone contour on each button (“–” for flat tone, “/” for rising tone, “∨” for falling then rising tone, “\” for falling tone) showed on the monitor, and the participant was instructed to press the button corresponding to the tone they heard. Before performing the adaptive 4AFC task in noise, participants were familiarized with the task by presenting the stimuli in the quiet condition. There were three types of stimuli in the quiet condition: original speech, resolved stimuli, and unresolved stimuli. Twenty stimuli were randomly selected from a pool of 36 stimuli for each stimulus type. For each participant, 60 stimuli played, corresponding to 15 stimuli for each lexical tone. The tone identification performance (mean ± standard deviation) was 93.89% ± 6.76%, 95.28% ± 6.52%, and 81.11% ± 9.16% for original speech, resolved stimuli and unresolved stimuli, respectively.

In separate blocks, the same adaptive procedure was used to test tone identification performance for each stimulus type. At each SNR, four stimuli with different Mandarin lexical tones were randomly played for four trials. If there were three or more correct identifications, the SNR was decreased; if there were less than three correct, the SNR was increased. The SNR step size was 5 dB for the first two reversals and 3 dB for the last six reversals. The task was ended when 8 reversals were reached. The SNR corresponding to 79.4% correct identification was defined as the averaged SNR of the last 6 reversals (Levitt, 1971). Each test procedure was performed twice for each participant, and the final estimated SNR was the averaged SNR of the two tests. Half of the participant began the test with resolved stimuli, and the other half began the test with unresolved stimuli.

Welch's *t*-test was used to assess whether the stimuli type (resolved and unresolved stimuli) had different effects on the estimated SNR.

### Electrophysiology

To determine whether adding noise for resolved and unresolved stimulus had different effects on the auditory brainstem neural temporal responses, the FFR to resolved and unresolved stimuli in quiet and in noise condition were recorded.

#### Electrophysiology—stimuli

The vowel /a/ with a rising tone (one of the acoustic stimuli used in the behavioral test) was used for the neurophysiological recording. Compared to the other tone contours, the rising tone has been shown to evoke the strongest FFR (Krishnan et al., 2004), therefore the rising F0 was used in the FFR experiments. The F0 of vowel /a/ ranged from 105 to 150 Hz. The vowel formants were: F1 676–760 Hz, F2 1160 Hz, F3 2470 Hz, F4 3900 Hz. The formants were estimated by linear predictive model in Praat (Boersma and Weenink, 2009). The unresolved stimuli version of the vowel /a/ was the same as in the behavioral test. However, for the resolved stimuli, the vowel /a/ was additionally high-pass filtered (150 Hz) to remove the vowel F0 in physiological experiment. Because there is no significant response at F0 for a few participants in the pilot test but a significant response at 2F0 (Sohmer et al., 1977; Aiken and Picton, 2008), the resolved stimuli were created by removing the F0 with a high-pass filter.

#### Electrophysiology—recording procedures

**Figure 1:**
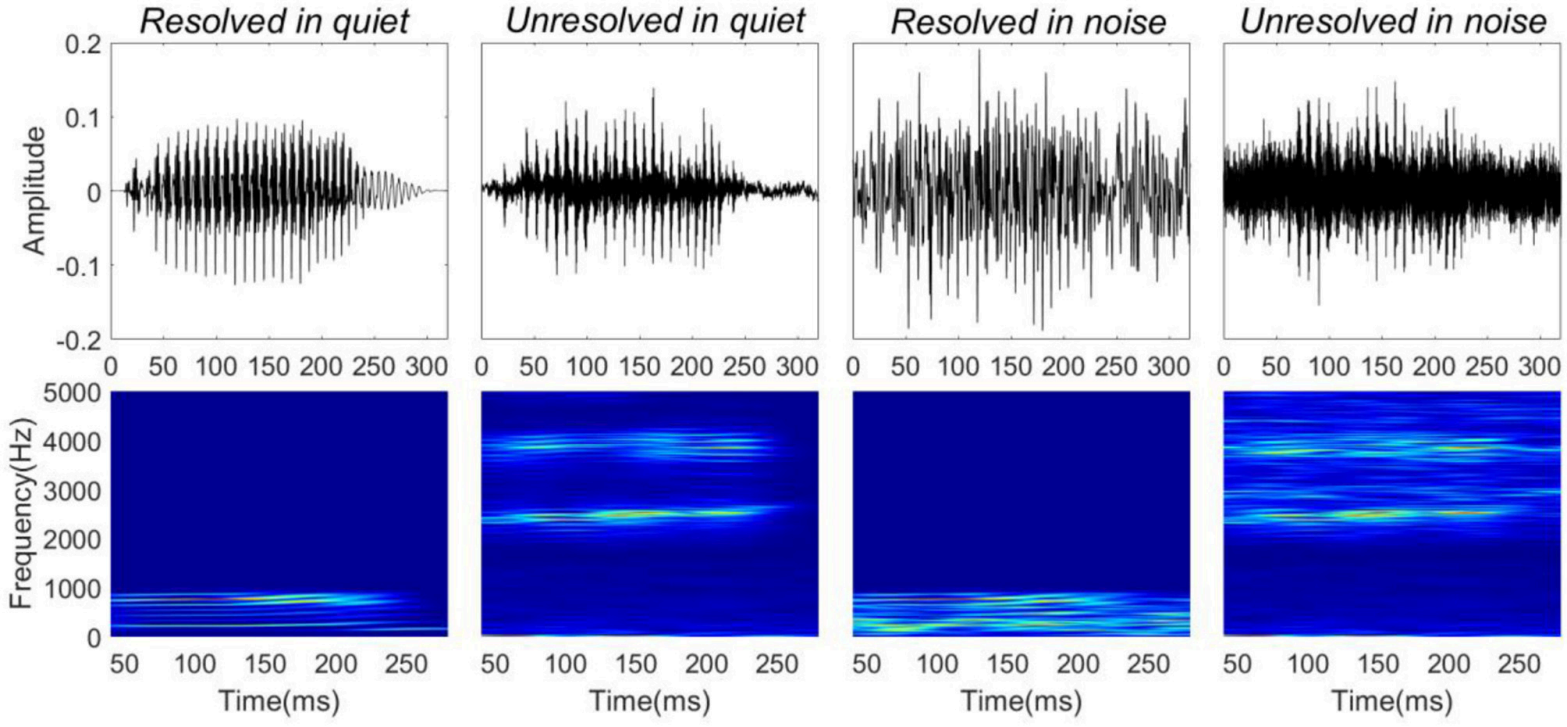
Stimuli waveforms (**top**) and spectrograms (**bottom**). From left to right, stimuli correspond to resolved stimuli in quiet, unresolved stimuli in quiet, resolved stimuli in noise, and unresolved stimuli in noise.

Four stimuli were presented for the FFR recordings: resolved stimuli in quiet, unresolved stimuli in quiet, resolved stimuli in noise (low-frequency speech-shaped noise) and unresolved stimuli in noise (high-frequency speech-shaped noise). The masking noise was the same as used in the behavioral test. In order to mask distortion products, a low intensity (45 dBA) filtered pink noise played back continuously for unresolved stimuli in both quiet and noise conditions. The four stimulus waveforms (top panels) and their corresponding spectrograms (bottom panels) are shown in Figure 1. The speech level was fixed at 65 dBA, and the SNR was 0 dB in the noise condition. The overall sound level was ~69 dBA in the noise condition. The duration of the stimuli was 300 ms, including 10 ms cosine function onset and offset ramps. The dBA scale was used to calibrate the speech level. The dB SPL of the resolved stimuli would be higher than 65, and the dB SPL of the unresolved stimuli would be similar to 65.

Participants sat comfortably in a sound-attenuated and electrically shielded booth while watching a subtitled silent movie of their choice. Participants were instructed to relax and allowed to sleep during the electroencephalography (EEG) recording. The stimuli were presented monaurally to the right ear through an electromagnetically shielded insert earphone (Etymotic ER2 in custom enclosure), while the left ear was plugged. The stimuli were presented in 240 blocks with alternating quiet and noise condition blocks. Each block contained 40 stimuli with alternating polarities. The stimuli were presented in a random order, generated separately for each participant. In noise condition, one long speech-shaped noise segment (40 s) was randomly selected at each block from five long speech-shaped noise segments. Continuous pink noise was used in both unresolved stimuli in quiet and in noise condition. To eliminate the possibility of specific phases of the stimulus coinciding with the EEG averaging time windows, the inter-stimulus interval was chosen on each trial from a uniform distribution between 80 and 120 ms (Zhu et al., 2013). The level of electrical cross-talk between the stimulus apparatus and the EEG sensors was tested by performing a recording when the insert-phone cables and transducers were mounted in the usual position, but the tubes were not inserted into the ear canal. We found no evidence of stimulus artifacts in the recorded response.

Continuous EEG was recorded differentially with a BioSemi Active2 system (BioSemi, Amsterdam, the Netherlands). Active electrodes were placed at Cz, the ipsilateral earlobe, contralateral earlobe, and the 7th cervical vertebra (C7). CMS/DRL electrodes were placed equidistant from FPz. This montage was the same as used by Smalt et al. (2012). The data were collected at a sample rate of 16.384 kHz, with an anti-aliasing low pass filter (which has a 5th order sinc response) with a −3 dB point at 3,200 Hz.

Offline, the continuous EEG signal at Cz was re-referenced to the average of the signals at the ipsilateral earlobe, the contralateral earlobe and C7. This reference configuration has been shown to have an optimal SNR for FFR (Krishnan et al., 2009, 2010b). Continuous EEG was then zero-phase filtered between 80 and 2000 Hz (6 dB/octave roll-off, 2,074 tap FIR filter) and segmented from −60 to 320 ms relative to stimulus onset. Epochs were baseline corrected to the mean of whole epoch and rejected when the amplitude of any sample exceeded ± 35 uV. The epochs corresponding to each stimulus polarity were added to derive the FFR_ENV_ or subtracted to derive FFR_TFS_ (Aiken and Picton, 2008), and the epochs corresponding to each stimulus condition were averaged. The FFR_ENV_ and FFR_TFS_ of each participant was the average of artifact-free trials (mean number of trials ± standard deviation (SD): 2083 ± 200) for each polarity (min 1,635, max 2,382 trials).

#### Electrophysiology—data analysis

All data were analyzed offline in MATLAB 2016b.

To evaluate how the averaged FFR_ENV_ of each participant reflected the time-varying periodicity, the running autocorrelogram was calculated. The autocorrelogram was constructed using a short-term autocorrelation function, which is mathematically similar to the frequency domain spectrogram (Krishnan and Plack, 2011). In the neurophysiology literature, the autocorrelogram is similar to the all-order interspike interval histogram that is used to represent spike timing features of the population of auditory nerve fibers (Cariani and Delgutte, 1996b). To calculate the autocorrelogram, the short-term autocorrelation function is first computed. This is the cross-correlation between the windowed response signal (30 ms time window shifted in 1 ms steps) and the copied response signal (shifted in 0.2 ms steps). The autocorrelogram (**Figure 4A**) is three dimensional, the horizontal axis represents the midpoint of the windowed response time and the vertical axis represents the time lags between the original signal and copied signal, i.e., pitch periods. Colors represent the magnitude of the correlation function at a given response time and corresponding autocorrelation lag time.

To quantify the robustness of periodicity encoded by the FFR_ENV_ in response to each stimulus, the peak autocorrelation value of each FFR_ENV_ response was calculated. The peak autocorrelation value of each FFR_ENV_ was defined as the average of the maximum autocorrelation values across the post-onset time period of 15–240 ms. The maximum autocorrelation value in each time bin was chosen from a lag time range limited to the stimulus period ± 2 ms.

To assess the degree of FFR_ENV_ synchrony in the spectral domain, the phase-locking value as a function of frequency and time was calculated for each participant and stimulus condition separately. The PLV measures the consistency of the phases of spectral components across trials. For each condition, the phase at each frequency and time was calculated with the short-term Fourier transform (80 ms time window, 2 ms steps) of the average or difference of a pair of single positive and negative polarity stimulus responses for FFR_ENV_ or FFR_TFS_, respectively. PLV for each stimulus condition was then calculated by the following equation (Tallon-Baudry et al., 1996; Mormann et al., 2000; Zhu et al., 2013).

$$\begin{array}{l} PLV=\sqrt{{\left[\frac{1}{N}\overset{N-1}{\underset{j=0}{\sum }}\sin\left(\varphi \;\left(t\right)\right)\right]}^{2}{+\left[\frac{1}{N}\overset{N-1}{\underset{j=0}{\sum }}\cos\left(\varphi \;\left(t\right)\right)\right]}^{2}} \end{array}$$

where N is the number of artifact-free trials. The number of artifact-free trials for each stimuli condition was balanced for each participant, as the baseline PLV changes based on the number of trials. Bootstrapping was used to estimate the baseline PLV for each participant, the phase at each time point and frequency was randomly shuffled and repeated 500 times, and the noise floor was defined as the 99% confidence intervals of PLV. The PLV curve at F0 (**Figure 5B**) for each participant was extracted by finding the maximum PLV within the stimulus F0 range (stimulus F0 ± 20 Hz).

To quantify the strength of FFR_ENV_ and FFR_TFS_ synchrony at F0 and harmonics, the peak PLVs at F0 and harmonics near to F1 (4th of F0) were calculated, respectively. The 4F0 at the grand average FFR_TFS_ was higher than the other harmonics and was selected to evaluate the FFR_TFS_ near to F1. The peak PLV at F0 and 4F0 were defined as the average of the PLV curve at F0 and 4F0 across a fixed time window (shaded area in **Figure 5B**) and was compared across stimulus conditions. The post-onset time range (15–240 ms) was chosen by finding the time range in which the grand average PLV curve at F0 was greater than the significance level generated by the bootstrapping method across all stimuli conditions. The stimulus frequency and duration might affect the response strength (Gockel et al., 2015).

To evaluate how closely the F0 curve extracted from the FFR_ENV_ followed the F0 in the stimulus, the stimulus-to-response correlation coefficient and F0 curve tracking error were calculated. The stimulus-to-response correlation coefficient was defined as the Pearson's correlation coefficient between the stimulus F0 curve and the response F0 curve. The stimulus F0 period extracted from the autocorrelogram of resolved stimulus in quiet by finding the maximum correlation coefficient within the corresponding time lag range (5–15 ms). The response F0 curve was extracted from the autocorrelogram for each condition by finding the peak correlation value at each time lag (stimulus period ± 2 ms). The F0 curve tracking error was calculated by taking the average of the absolute differences between the stimulus and the response F0 at each time point (Song et al., 2008).

### Statistical tests

The FFR_ENV_ F0 strength was evaluated in the temporal domain by the peak autocorrelation value and in the spectral domain by the peak PLV at F0 for each stimulus condition. Statistical significance was assessed using a two-way repeated measures analysis of variance (ANOVA), with participants included as a random factor. The ANOVA determined whether the resolvability (resolved and unresolved stimulus) and noise (quiet and noise condition) had effects on the FFR_ENV_ F0 strength in the temporal and spectral domains. *Post-hoc* Tukey tests were used to evaluate whether there was a significant difference between the robustness of pitch encoded by the FFR_ENV_ in response to stimuli with different resolvability and noise condition. To measure the effect of noise on the strength of peak PLV at 4F0 for the resolved stimuli, a paired *t*-test was used. To measure the noise effect on the F0 tracking as reflected by FFR_ENV_, a two-way repeated measures ANOVA used to test whether the factors of resolvability and noise had effects on the stimulus-to-response correlation coefficient and F0 curve tracking error.

To explore whether there was a correlation between the behavioral task performance and the FFR_ENV_ F0 strength to unresolved stimuli, Spearman's rank coefficient r_s_ was calculated. The statistical significance level was defined as *p* < 0.05.

The behavioral performance was the tone correct percentage in quiet and at an SNR of 0 dB. The percentage correct was calculated by dividing the number of correct responses by the number of speech sounds presented. Due to the adaptive procedure used to test tone identification in noise, the number of speech sounds presented for each participant was different. Three participants who had < 10 stimuli presented at 0 dB SNR were excluded from this analysis.

## Results

### Behavioral test

**Figure 2:**
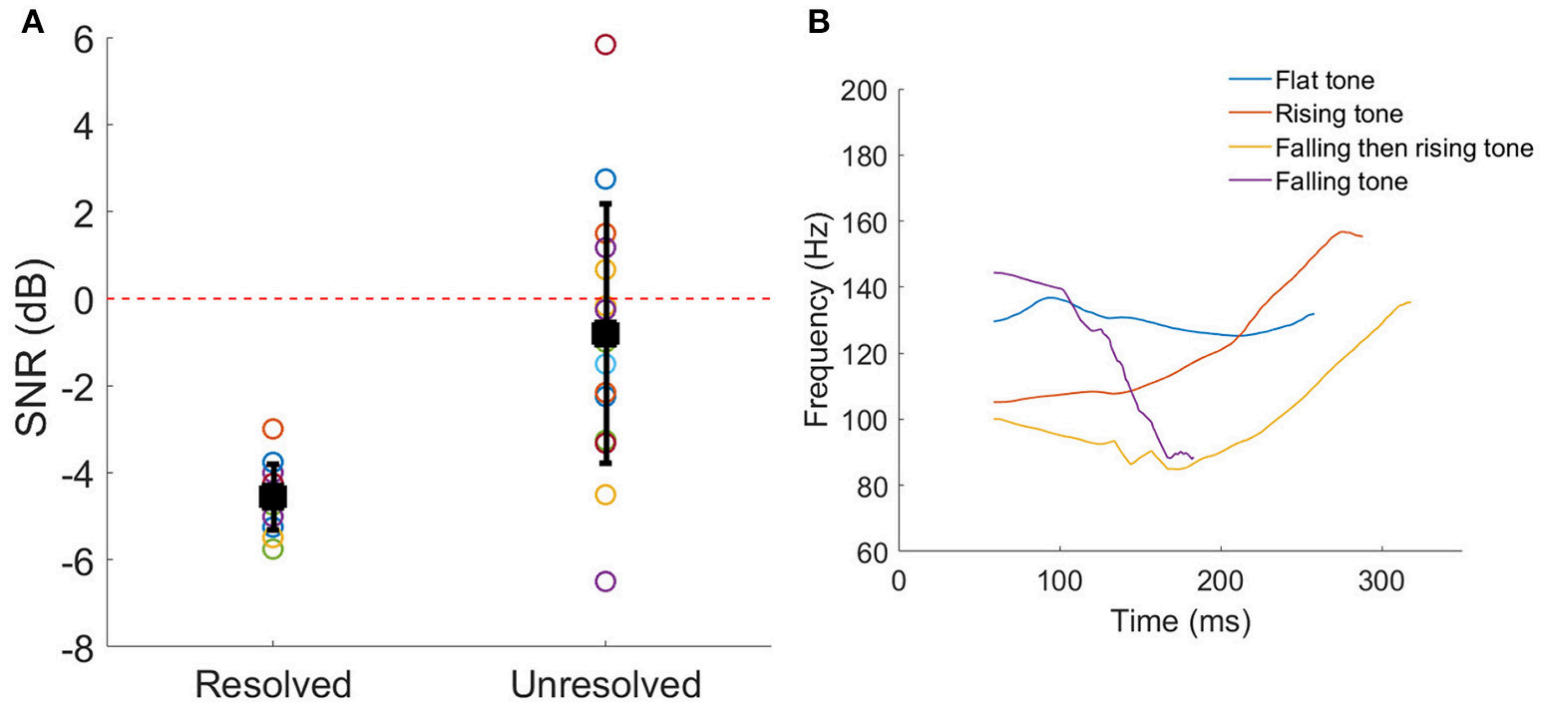
**(A)** The SNRs corresponding to 79.4% correct Mandarin tones identification for each participant. The black squares represent the mean SNR, and the line bars represent the SD. The red dashed line represents the SNR = 0 dB which was used in the FFR recording. **(B)** The four F0 contours of a vowel /a/. The color represents the different tone.

The SNRs corresponding to 79.4% correct identification of the Mandarin tones for each participant are shown in Figure 2A. Welch's *t*-test indicated that the 79.4% correct SNR for resolved stimuli was significantly lower (better performance) than unresolved stimuli (*t* = −4.60, *p* < 0.001).

### FFR results

**Figure 3:**
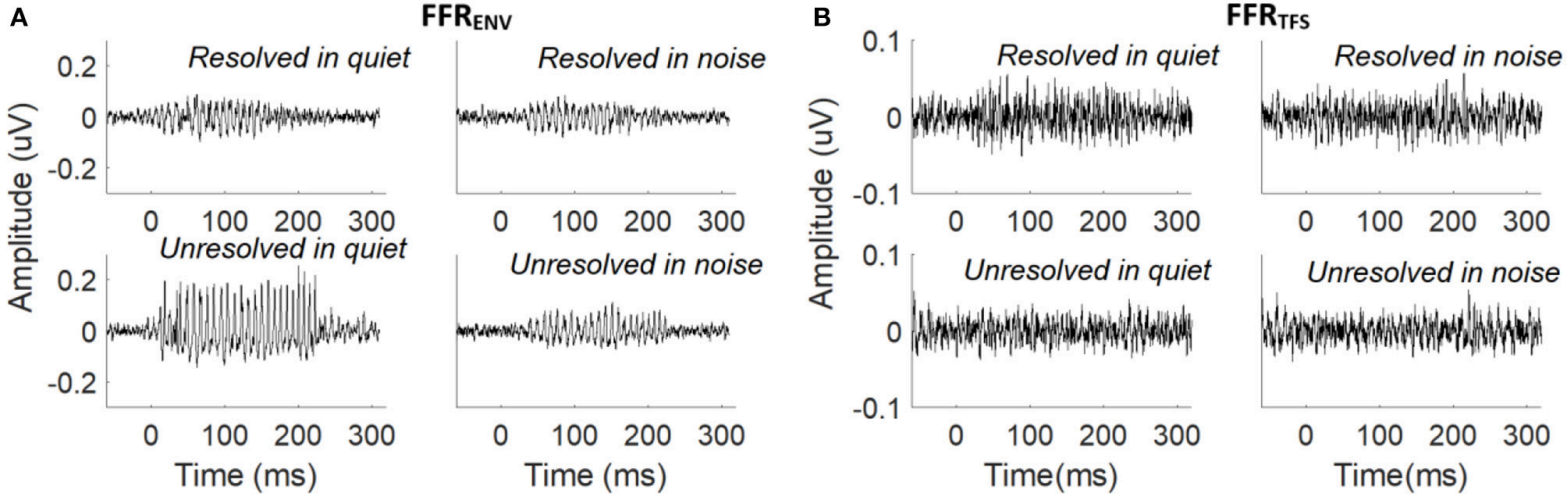
**(A)** Grand average FFR_ENV_ waveforms to the resolved stimuli in quiet and noise (top panel) and unresolved stimuli in quiet and noise (bottom panel). **(B)** Grand average FFR_TFS_ waveforms to the resolved stimuli in quiet and noise (top panel) and unresolved stimuli in quiet and noise (bottom panel).

The grand average FFR_ENV_ (Figure 3A) and FFR_TFS_ (Figure 3B) waveforms to the resolved stimuli in quiet and noise (top panels), and unresolved stimuli in quiet and noise (bottom panels) are displayed. The FFR_ENV_ waveforms show clear periodicity in all conditions. The amplitudes in the pre-stimulus baseline (signal before 0 ms) for all conditions of FFR_ENV_ are similar, suggesting that the continuous noise presented during the noise condition trials did not contribute to the response. The FFR_TFS_ to the four stimuli conditions are noisy and the amplitudes of FFR_TFS_ are lower than FFR_ENV_.

**Figure 4:**
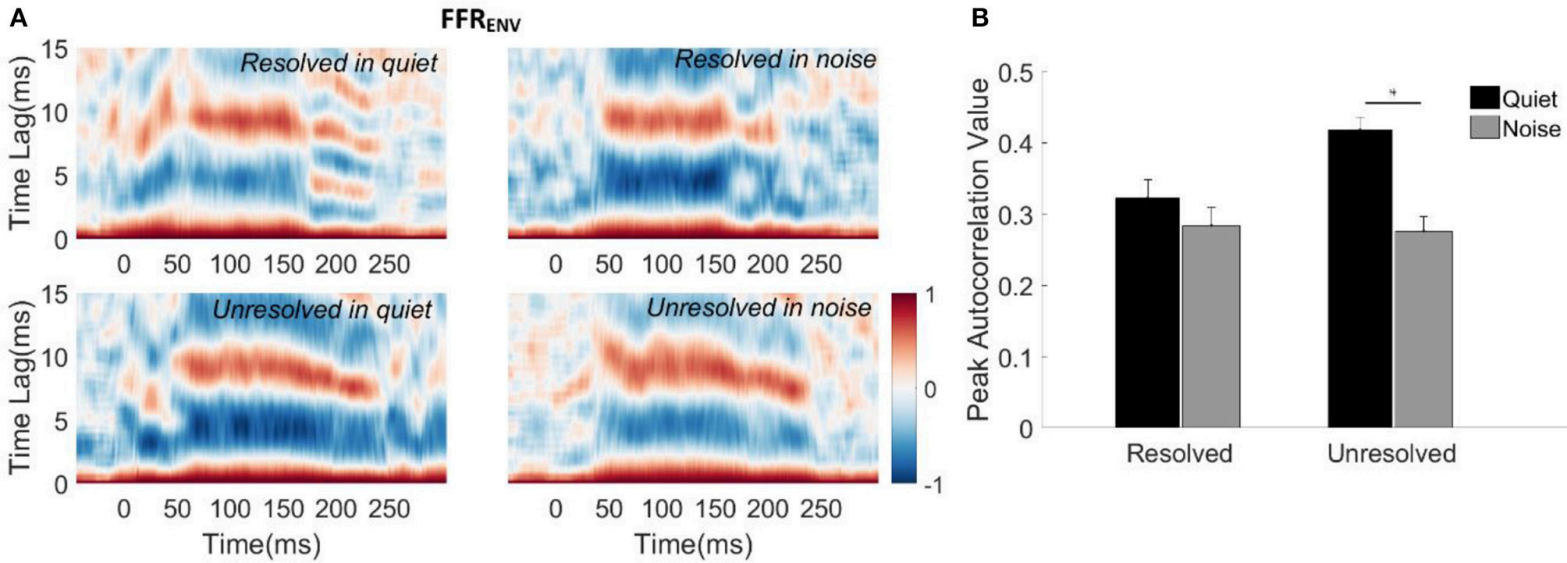
**(A)** Autocorrelograms of grand average FFR_ENV_ waveforms for each stimulus condition. The horizontal axis represents the midpoint in each 30 ms time bin, the vertical axis represents the time lags between the time windowed signal with its copy signal, and the color represents the strength of correlation (red is maximum). **(B)** The peak autocorrelation value averaged across participants for each stimulus condition. The black bars represent the stimulus in the quiet condition, and gray bars represent the stimulus in noise condition. Error bars represent one standard error. The significant difference of p < 0.001 is indicated by the asterisk.

To evaluate how well the FFR_ENV_ coded periodicity, the autocorrelograms of the grand average FFR_ENV_ waveforms for each stimulus condition were calculated and are displayed in Figure 4A. The peak correlation bands occurred at time lags from 9.5 to 7 ms for all of the four stimuli. These lags correspond to the stimulus F0 periods (9.5–6.5 ms, 105–153 Hz). However, the autocorrelation values corresponding to the F0 for the resolved stimuli (Figure 4A, top panels) started decreasing from 180 ms, and there was a weak response at the time lag equal to half the stimulus period (4–3.5 ms) for resolved stimuli in the quiet condition (Figure 4A, top left panel). This suggests that there may have a response to 2F0 for the resolved stimuli in quiet for some participants.

To quantify the robustness of FFR_ENV_ coded periodicity, the peak autocorrelation value was extracted from the autocorrelogram of each condition for each participant. The mean peak autocorrelation values across participants are displayed in Figure 4B. To explore whether the resolvability of the stimulus and noise had effects on the peak autocorrelation value of FFR_ENV_, a two-way repeated measures ANOVA (with resolvability and noise as fixed factors, participants as a random factor) were conducted. The main effect of resolvability [*F*_(1,51)_ = 10.67, *p* = 0.002] and noise [*F*_(1,51)_ = 45.46, *p* < 0.001] on the peak autocorrelation value of FFR_ENV_ were significant, and there was a significant interaction effect [*F*_(1,51)_ = 14.90, *p* < 0.001] between the two factors. *Post-hoc* Tukey multiple comparisons indicated that the peak autocorrelation value for unresolved stimuli in quiet was significantly greater than for unresolved stimuli in noise (*t* = 7.5, *p* < 0.001), and resolved stimuli in quiet (*t* = 5.04, *p* < 0.001) and in noise (*t* = 7.08, *p* < 0.001). There was no significant difference between the peak autocorrelation values for resolved stimuli in quiet and in noise.

**Figure 5:**
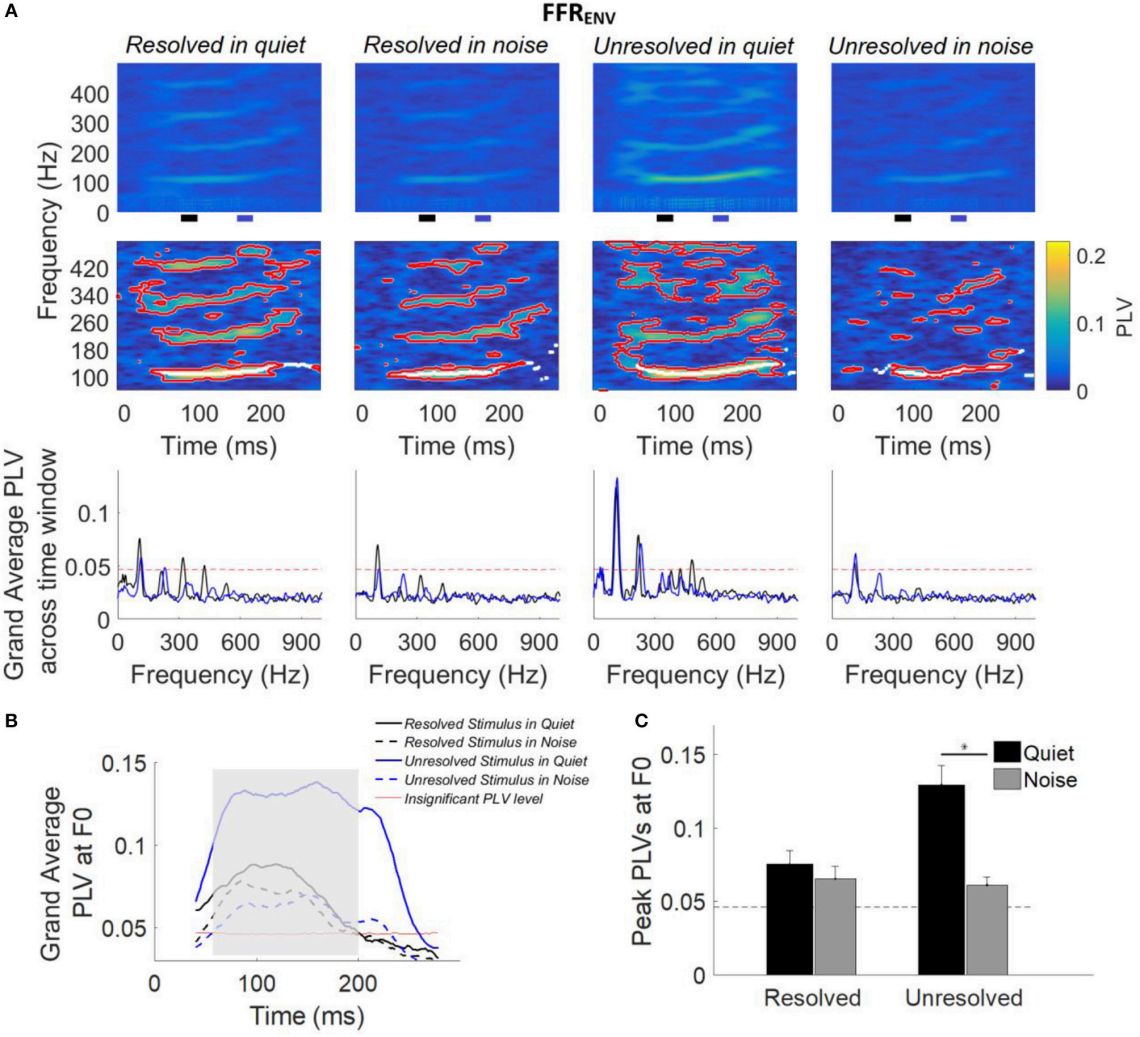
**(A)** Grand average FFR_ENV_ PLVs (top panels) and FFR_ENV_ PLVs from a representative participant (middle panels) as a function of time and frequency for each condition. In the middle panel, PLVs that were significantly higher than the bootstrapped noise distribution are enclosed by red lines, and PLV curves at F0 (the maximum PLV at each time point) are shown in white lines. The horizontal axis represents the midpoint of the 80 ms analysis window, the vertical axis represents the frequency, and the color represents the PLV at each time and frequency (yellow is largest). The grand average FFR_ENV_ PLV across participants (bottom panel) as a function of response frequency at two different time slides. The black and blue line represents the averaged PLV from 80 to 100 ms (black bar) and from 160 to 170 ms (blue bar). The black and blue bar showed in the bottom of the top panel. **(B)** Grand average PLV curves at F0 for each stimulus condition. Responses above the red line are significant (*p* < 0.01). The shaded area represents when PLV is significant at F0 for all four conditions. The time represents the midpoint of the 80 ms time window. **(C)** Peak PLV at F0 averaged across participants for each stimulus condition. Error bars represent one standard error. Responses above the dashed back line are significant (*p* < 0.01). The significant difference of p < 0.001 is indicated by the asterisk.

Figure 5 shows the grand average FFR_ENV_ PLVs across participants (top panel) and FFR_ENV_ PLVs from a representative participant (middle panel), and grand average FFR_ENV_ PLVs across participants as a function of response frequency (bottom panel) for each stimulus condition. The grand average FFR_ENV_ PLVs show that FFR_ENV_ is strongest at the stimulus F0 and weaker at the harmonics for all conditions. For resolved stimuli, the FFR_ENV_ PLVs is visible for the second, third and fourth harmonics of stimuli.

For each participant (data from a representative participant is shown in the bottom row of Figure 5A), bootstrapping was used to generate a distribution of PLVs forming the noise floor, and PLVs significantly larger than the noise floor (*p* < 0.01) are enclosed by a red outline (Figure 5A, middle panel). The white lines (Figure 5A, middle panel) show the maximum PLV in the stimulus F0 range (± 20 Hz) for each time point, and form the PLV curve at F0.

The grand average FFR_ENV_ PLV across two different time segments are shown in Figure 5 (bottom panel). The black and blue line represents the grand average FFR_ENV_ PLV across 80–90 ms (black bar in the top panel) and 160–170 ms (blue bar in the top panel), and the F0 was shifted from 108 to 116 Hz. The FFR_ENV_ PLV at F0 was higher than the noise floor (red dashed line) for all stimuli conditions, and FFR_ENV_ PLV at harmonics was also higher than the noise floor only for stimuli in quiet condition.

The grand average PLV curves at F0 are displayed in Figure 5B. The average PLV curves at F0 for stimuli in quiet (Figure 5B, solid line) are greater than that in noise condition (Figure 5B, dashed line). The PLV curves at F0 which are significantly different from baseline in all four conditions are indicated by the shaded area.

To quantify the strength of FFR_ENV_ synchrony in the spectral domain, the peak PLV at F0 was calculated for each participant. The average peak PLV across participants as a function of the different stimulus conditions is shown in Figure 5C. A two-way repeated measures ANOVA (with resolvability and noise as fixed factors and participants as a random factor) was performed to examine whether the resolvability of stimuli and noise have effects on the peak PLV at F0. ANOVA revealed that both the resolvability [*F*_(1,51)_ = 20.49, *p* < 0.001] and noise [*F*_(1,51)_ = 50.87, *p* < 0.001] had significant effects on the peak PLV at F0, and that the interaction between the two factors on the peak PLV at F0 was also significant [*F*_(1,51)_ = 28.27, *p* < 0.001]. *Post-hoc* Tukey multiple comparisons found that the peak PLV at F0 of the unresolved stimuli in quiet was significantly (*p* < 0.001) larger than the other three stimuli conditions. The peak PLV at F0 for unresolved stimuli in quiet was significantly (*t* = 0.068, *p* < 0.001) stronger than in noise, but with no significant difference for resolved stimuli in quiet and noise conditions.

**Figure 6:**
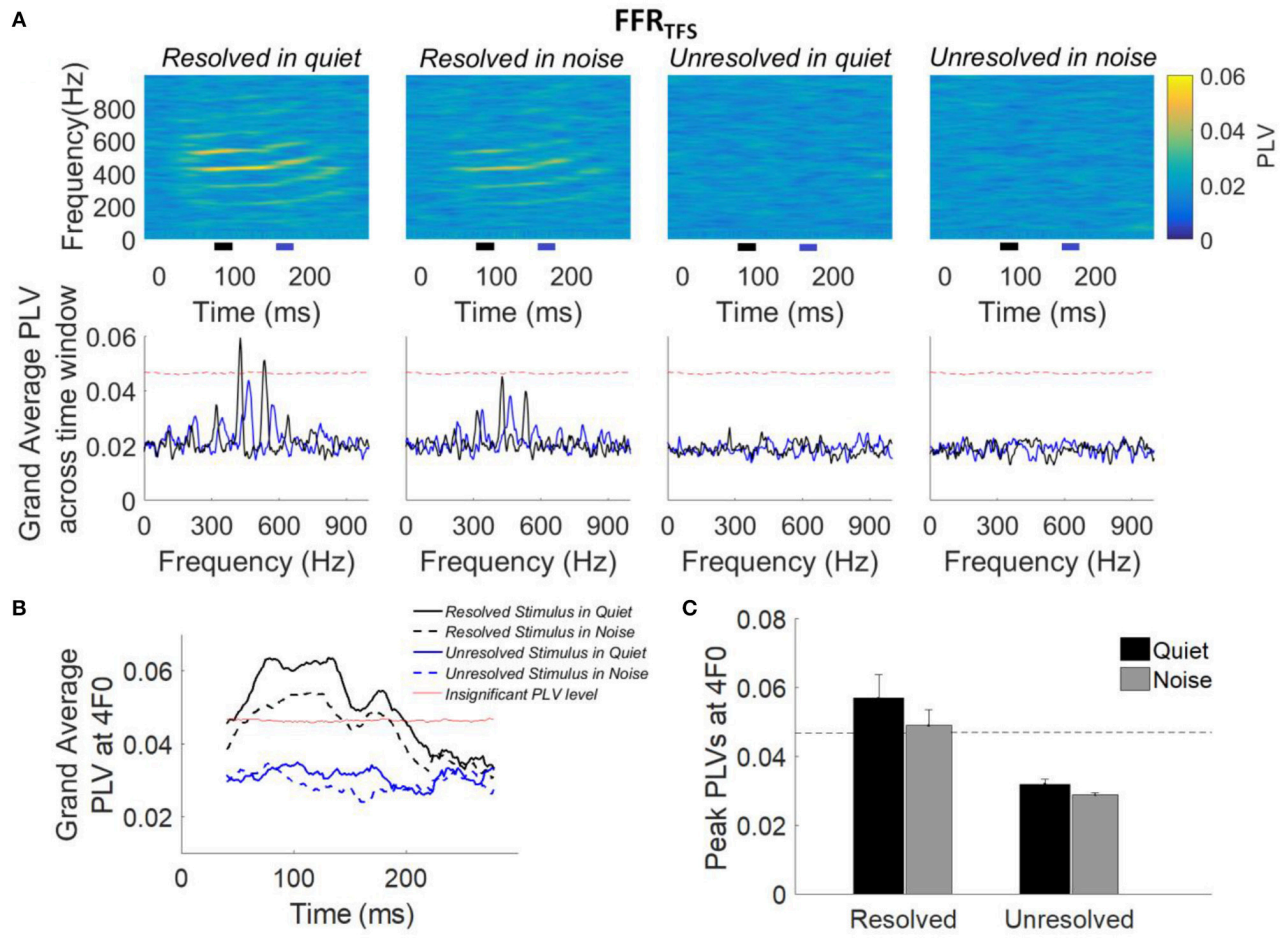
**(A)** Grand average PLVs of FFR_TFS_ (top panel) as a function of time and frequency for each condition. The horizontal axis represents the midpoint of the 80 ms time window used for spectral phase calculation, the vertical axis represents the frequency, and the color represents the magnitude of PLV at each time and frequency. The grand average FFR_TFS_ PLV across participants (bottom panel) as a function of response frequency at two different time slides. The black and blue line represents the averaged PLV from 80 to 100 ms (black bar) and from 160 to 170 ms (blue bar). The black and blue bar showed in the bottom of the top panel. **(B)** Grand average PLV curves at 4F0 for each stimulus condition. Responses above the red line are significantly greater than the noise level (*p* < 0.01). The time represents the midpoint of the 80 ms analysis window. **(C)** Peak PLVs at 4F0 averaged across participants for each stimulus condition. Error bars represent one standard error. Responses above the dashed back line are significant (*p* < 0.01).

The grand average PLVs of FFR_TFS_ (top panel) and the grand average PLVs of FFR_TFS_ across time segments (bottom panel) to each stimulus condition are shown in Figure 6A. Compared to the PLVs for the unresolved stimuli, PLVs of FFR_TFS_ can be seen at the fourth and fifth harmonics in quiet and in noise (Figure 6A, top panels) for the resolved stimuli. The grand average FFR_TFS_ PLVs across 80–90 ms (black line) and 160–170 ms (blue line) are showed in Figure 6A (bottom panel), and the 4F0 is shifted from 432 to 464 Hz. The clear peaks showed at fourth and fifth harmonics in the grand average FFR_TFS_ PLVs only for resolved stimuli, and are higher than the noise level in quiet condition. To quantify the PLV at the harmonics, the grand average PLV curves at 4F0 were computed and are displayed in Figure 6B. The average PLV at 4F0 for the resolved stimuli in quiet (Figure 6B, solid black line) is greater than in the noise condition (Figure 6B, dashed black line). However, the average PLV at 4F0 for the unresolved stimuli was not significantly higher than the bootstrapped noise level. To quantify the strength of FFR_TFS_ synchrony in the spectral domain, the peak PLV at 4F0 was calculated. The mean peak PLV at 4F0 as a function of the stimulus condition is shown in Figure 6C. A two-way repeated measures ANOVA (with resolvability and noise as fixed factors and participants as a random factor) was performed to examine whether the resolvability of stimuli and noise have effects on the peak PLV at 4F0. ANOVA revealed that the resolvability [*F*_(1,51)_ = 37.93, *p* < 0.001] had significant effects on the peak PLV at 4F0.

**Figure 7:**
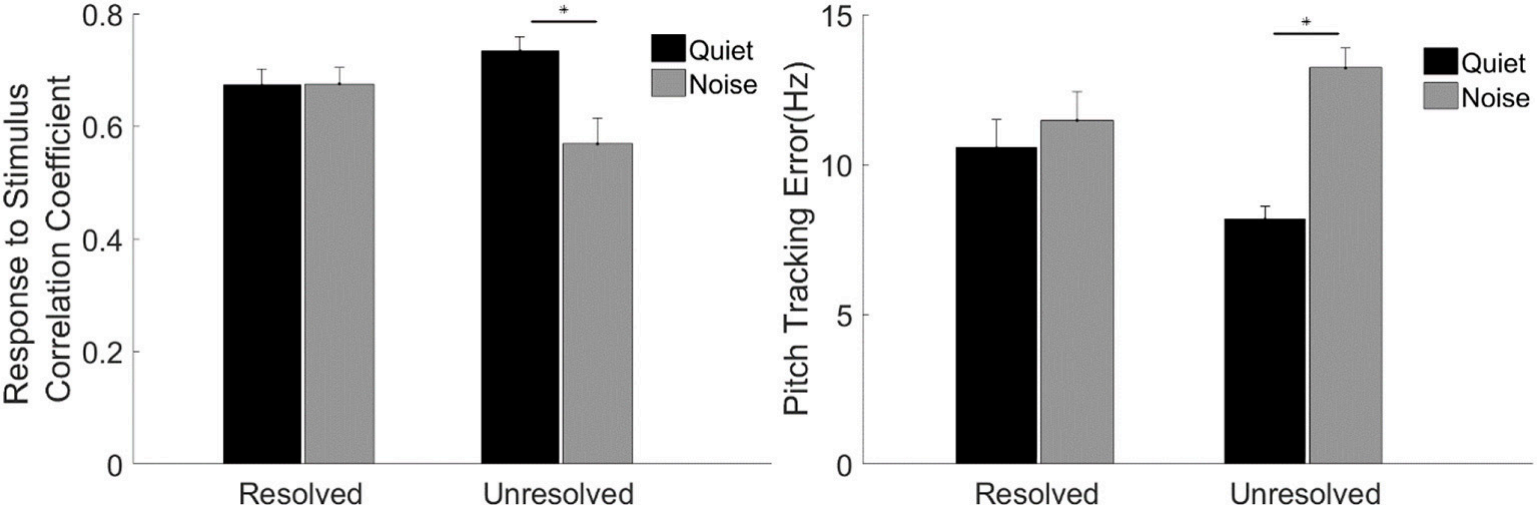
The stimulus-to-response correlation coefficient (**left**) and F0 curve tracking error (**right**) averaged across participants for each stimulus condition. The black and gray bars represent the response in quiet and in noise condition, respectively. Error bars represent one standard error of the mean. The significant difference of p < 0.001 is indicated by the asterisk.

To evaluate the F0 curve tracking accuracy encoded by the FFR_ENV_, the stimulus-to-response correlation coefficient and pitch tracking error averaged across participants are displayed in Figure 7. A two-way repeated measures ANOVA (with resolvability and noise as fixed factors, participants as a random factor) was performed to examine whether the resolvability of stimuli and noise have effects on the stimulus-to-response correlation coefficient and F0 curve tracking error. Two-way ANOVA showed that the noise [*F*_(1,51)_ = 7.64, *p* = 0.008] had significant effect on the stimulus-to-response correlation coefficient, and the interaction effect of two factors on the stimulus-to-response correlation coefficient was also significant [*F*_(1,51)_ = 8.11, *p* = 0.006]. *Post-hoc* test showed that the stimulus-to-response correlation coefficient of FFR_ENV_ to unresolved stimuli in quiet was significantly (*t* = 3.97, *p* = 0.001) higher than in noise. ANOVA showed that the main effect of noise was significant [*F*_(1,51)_ = 28.40, *p* < 0.001), and the interaction effect of two factors was significant [*F*_(1,51)_ = 13.66, *p* = 0.001) on the pitch tracking error. *Post-hoc* Tukey test showed that the pitch tracking error of unresolved stimuli in quiet was significantly (*t* = −6.38, *p* < 0.001) lower than in noise, and there is no significant difference between the resolved stimuli in quiet and in noise.

To explore the relationship between the two measurements of FFRenv peak autocorrelation value and peak PLV at F0, the Spearman's correlation was calculated. The relationship between the peak autocorrelation value and peak PLV at F0 of all stimuli conditions were shown in Figure 9, and the peak autocorrelation value was significantly increased with peak PLV at F0.

### The correlation between the behavior correct tone identification with FFR index

**Figure 8:**
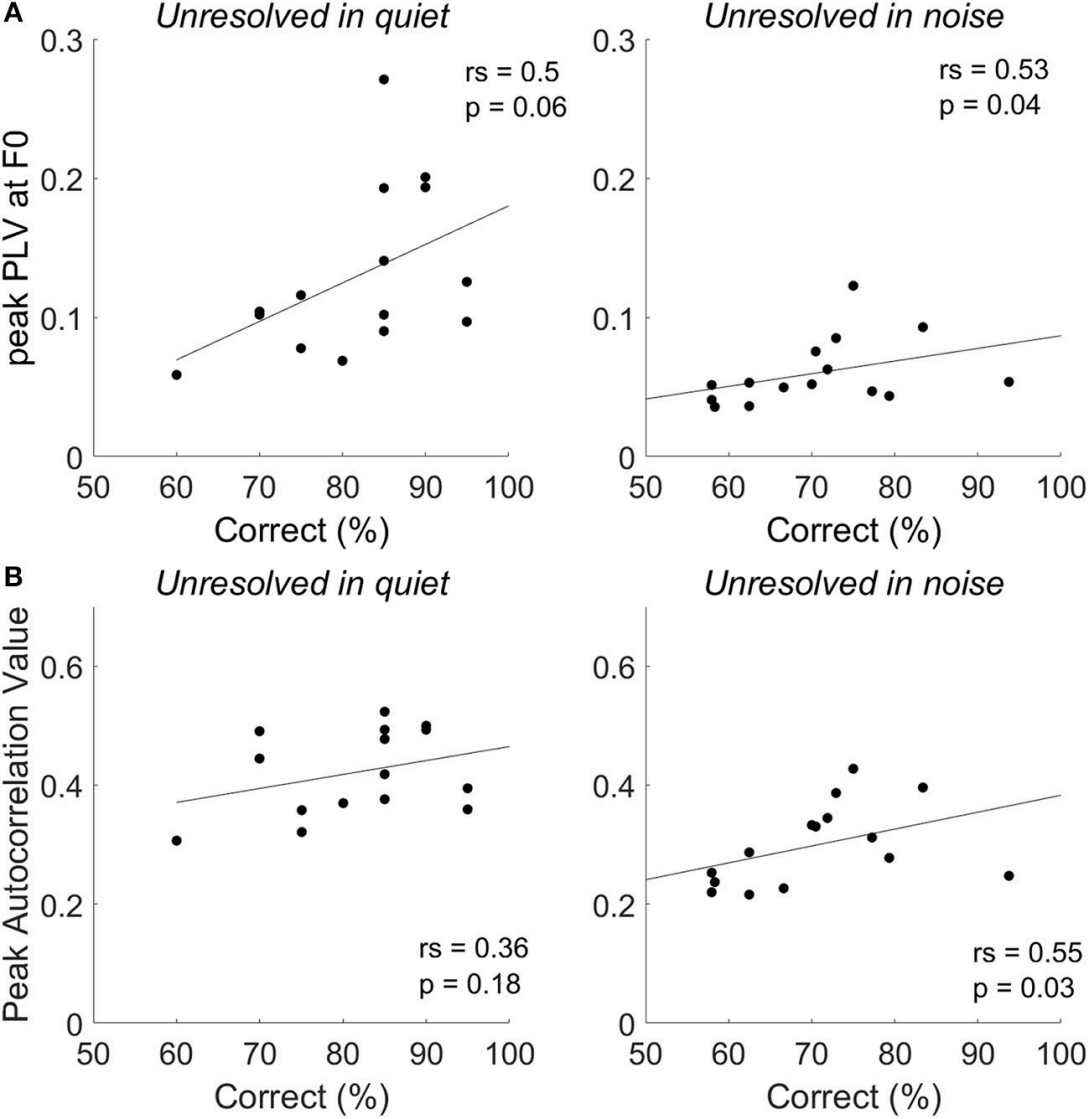
The correlation between tone identification performance and the peak PLV at F0 **(A)** and peak autocorrelation value **(B)** for unresolved stimuli in quiet and in noise (0 dB SNR).

To explore whether the F0 strength reflected by the FFR_ENV_ was correlated with tone identification performance in quiet and in noise (0 dB SNR), a Spearman's correlations were performed (Figure 8). For resolved stimuli, both FFR_ENV_ F0 strength and the PLV at harmonics would contribute to the tone identification performance. However, either the FFR_ENV_ F0 strength or the PLV at harmonics is not correlated with the behavior tone identification performance for both resolved stimuli in quiet and in noise. The correlation between the correct tone identification percentage and the peak PLV at F0 (Figure 8A, right panel) and peak autocorrelation value (Figure 8B, right panel) of unresolved stimuli are significant at SNR of 0 dB, but not in quiet. The results suggest that FFR_ENV_ F0 strength was related to the tone identification performance in noise.

**Figure 9:**
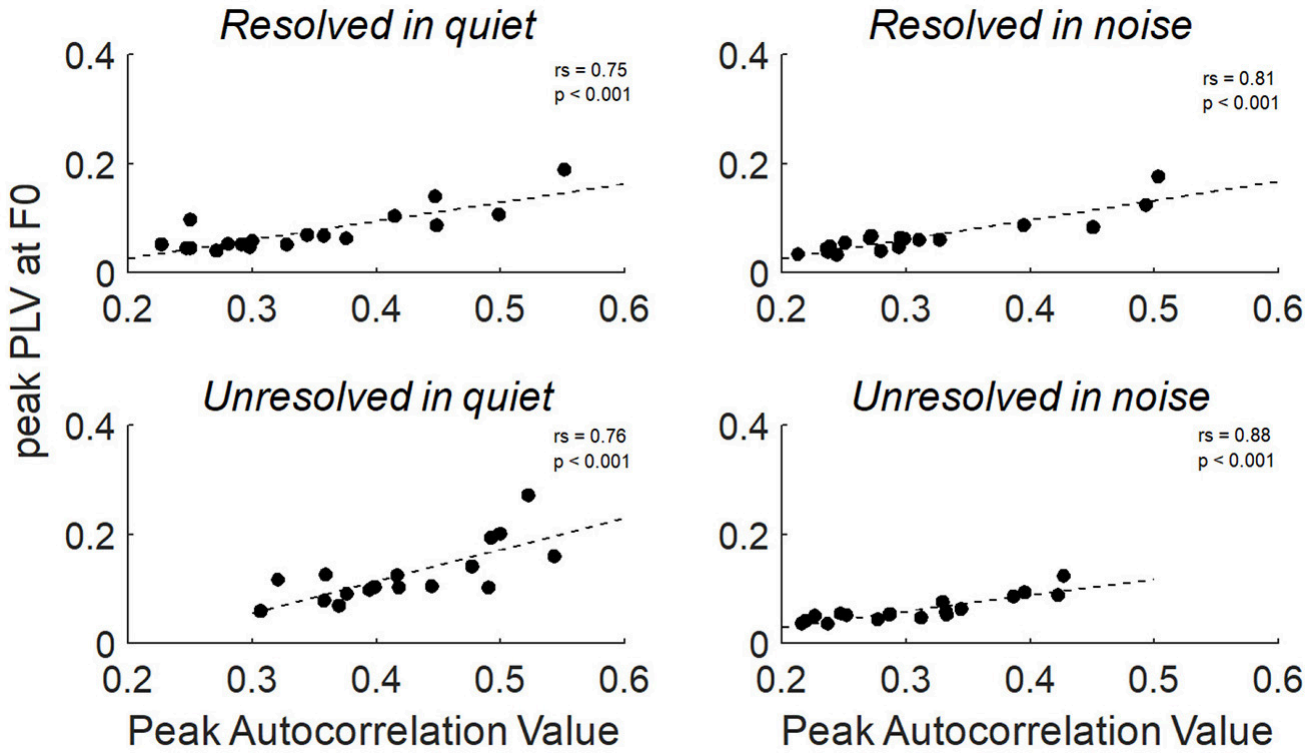
The correlation between the peak PLV at F0 and peak autocorrelation value for resolved stimuli in quiet and in noise (**top**) and unresolved stimuli in quiet and in noise (**bottom**).

## Discussion

In this study, we aimed to evaluate subcortical neural temporal information as reflected by FFR in response to resolved and unresolved harmonics of a vowel sound with dynamic F0 contour in quiet and in speech-shaped noise. The FFR_ENV_ and FFR_TFS_ were used to evaluate the subcortical neural representation of the envelope and the temporal fine structure, respectively. The FFR_ENV_ F0 strength was evaluated by the peak autocorrelation value and the peak PLV at F0. For the FFR_ENV_, we found that both evaluation methods showed that the F0 strength in quiet was significantly stronger than in noise, but only when the stimuli contained only unresolved harmonics. For the FFR_TFS_, we evaluated the peak PLV at 4F0, and found a significantly stronger response for resolved stimuli but not for unresolved stimuli. Overall, the results suggest that the F0 encoding strength of FFR_ENV_ to stimuli with dynamic F0 contours containing only resolved harmonics is more noise robust than for stimuli containing only unresolved harmonics.

### Tone identification performance

In the current study, the SNR at 79.4% correct performance level for resolved stimuli was significantly lower than for unresolved stimuli, suggesting that the resolved harmonics in speech play a key role in tone identification in noise. Our results are similar to those found by Kong and Zeng (2006) and Luo and Fu (2006), who found that the lower harmonics contributed most strongly to tone identification in noise. However, Liu et al. (2014) showed a different result, whereby tone identification performance for stimuli containing higher harmonics (higher than the 3rd harmonic) was better than for lower harmonics (lower than the 3rd harmonic) in noise. This discrepancy may be explained by the different filters used to create the stimuli in each study. In our study, harmonics lower than the 10th were defined as resolved, and the number of harmonics greater than the 10 was defined as unresolved (Houtsma and Smurzynski, 1990; Bernstein and Oxenham, 2003). The resolved stimuli in our study included more individual harmonics, which would generate a stronger response at F0 than the stimuli only containing three harmonics. The ability of the auditory system to extract the F0 from a complex tone including resolved harmonics is more noise-robust than from a complex tone including unresolved harmonics (Gockel et al., 2006).

### FFR results

In our study, we first compared the FFR_ENV_ F0 strength to resolved and unresolved stimuli. We found that F0 strength was significantly higher for unresolved compared to resolved harmonics in the quiet condition, both in the spectral and time domains. In the spectral domain, our findings are consistent with the studies of Jeng et al. (2011) and Zhu et al. (2013), and suggest that FFR_ENV_ F0 strength is mostly produced by the peripheral auditory system responding to the unresolved harmonics in the stimulus. In the time domain, however, our findings are opposite to those of Krishnan and Plack (2011), who found that the peak autocorrelation value of FFR to the resolved harmonics of complex tones (5 harmonics) was significantly larger than for the unresolved harmonics. One possible reason that we found a larger FFR_ENV_ F0 strength in response to unresolved stimuli compared to resolved stimuli is that the number of harmonics (> 28) in the unresolved stimuli in our study was greater than that in the resolved stimuli, whereas Krishnan and Plack (2011) used an equal number of harmonics in their resolved and unresolved stimuli. Stimuli with a greater number of harmonics present may generate a stronger response at F0 (Kaernbach and Bering, 2001).

We found that the FFR_ENV_ F0 strength to resolved stimuli was more noise robust than to unresolved stimuli when speech-shaped noise was used to balance the masking effect. Our results were similar to those found by Laroche et al. (2013) using white noise. For resolved stimuli (in which F0 strength was not affected by noise), our result was in agreement with the study by Zhu et al. (2013), which showed that addition of Gaussian noise had no significant effect on the PLV at F0 to resolved harmonics of a complex tone. For unresolved stimuli, our results were also similar to those found by Laroche et al. (2013), who showed that the spectral magnitude at F0 of FFR_ENV_ to stimuli dominated by unresolved harmonics was significantly lower in noise compared to quiet.

It has been suggested that the FFR_ENV_ reflects encoding of the envelope of stimuli for both resolved and unresolved stimuli (Skoe and Kraus, 2010; Shinn-Cunningham et al., 2017). For resolved stimuli, the FFR_ENV_ might be related to the envelope in each auditory filter (Skoe and Kraus, 2010; Shinn-Cunningham et al., 2017), where the output of the auditory filter beats at F0 when it is positioned between two harmonics (Krishnan and Plack, 2011). This theory was supported by the study of Gockel et al. (2011), who showed that the first peak of the spectrum of the FFR_ENV_ corresponded to the stimulus F0, and did not change when the individual harmonics of a complex tone were shifted up or down. For unresolved stimuli, the FFR_ENV_ is related to neural responses to the interaction of multiple harmonics in a single auditory filter. In background noise, the SNR which reaches the auditory filter corresponds to the amount of noise and signal in the input sound stimulus (Moore, 2012). Because the auditory filter bandwidth is narrow at lower frequencies, the within-band SNR of resolved stimuli would be higher than for unresolved stimuli.

In our study, we found strong PLV responses at the harmonics (4F0) in the PLVs of FFR_TFS_ for the resolved stimuli only. This result is consistent with studies showing that the FFR reflects neural responses to vowel formants (Krishnan, 2002; Krishnan et al., 2004; Aiken and Picton, 2008). We found no significant PLV in FFR_TFS_ to the unresolved stimuli, in agreement with the idea that the FFR_TFS_ only reflects neural phase-locking to frequencies lower than 1,500 Hz (Aiken and Picton, 2008).

For unresolved stimuli, we found that the moderate correlation between tone identification performance and FFR_ENV_ F0 strength was significant in noise (0 dB SNR), but not in quiet. This was the case when F0 strength was measured in both the temporal domain (evaluated by the peak autocorrelation measure) and in the spectral domain (evaluated by peak PLV at F0). However, these results should be approached with caution: in our behavioral tasks we used an adaptive procedure which was designed to determine the SNR at which 79.4% performance was achieved. To perform correlations with the FFR_ENV_ F0 strength, we subsequently calculated the performance level (% correct) at 0 dB SNR. This noise level was just one point on the adaptive track and was therefore presented to each participant a different number of times. Nevertheless, the results are consistent with those of Coffey et al. (2017a), who examined FFR to a syllable /da/ in quiet, and found that F0 strength was not correlated with speech in noise performance. Together, the results suggest that FFR_ENV_ F0 strength to stimuli in quiet might not predict the performance in noise. One possible reason is that the FFR was generated from several subcortical nuclei sources, and the potential at Cz might be affected by differences in individual brain geometries (Coffey et al., 2016a).

For resolved stimuli, however, there was no significant correlation between F0 strength of FFR_ENV_ with the tone identification performance. One possible reason is that the harmonics of FFR_TFS_ was also related with tone identification, suggested by result whereby tone identification was nearly perfect when only depending on the individual harmonics in quiet (Liu et al., 2014).

In our study, the FFR_ENV_ peak autocorrelation value was significantly and positively correlated with the peak PLV at F0 across participants. The two methods in our study are both spectral methods that disregard the absolute magnitude of the response. The difference is that the autocorrelogram uses the temporal average before extracting phase information whereas the PLV extracts phases individually then combines the information. A pronounced difference would have been observable if there was only partial phase locking across trials. The similarity in results produced by the two methods may be evidence that there is little variation in response morphology of the FFR_ENV_.

## Conclusions

We examined the neural coding of dynamic F0 contours in Mandarin speech sounds. The FFR_ENV_ F0 strength to speech sounds with resolved harmonics was more noise robust than when the speech sounds contained unresolved harmonics. However, for unresolved stimuli, people with good tone identification had stronger FFR_ENV_ F0 strength in noise.

Our results suggested that the resolved harmonics play an important role in tone identification, and have implications for people with hearing loss who use a cochlear implant. Most current cochlear implants sound-coding strategies do not encode the temporal fine-structure of sounds, leaving only envelope cues and place cues to convey pitch-related information. Our results suggest that encoding temporal aspects of pitch (by controlling pulse timing for example) may be important for cochlear implant users who need to understand tonal languages.

## Author contributions

FP, HI-B, CM, and WH designed experiments. FP performed experiments and collected data. FP, HI-B, CM, and DM analyzed data and interpreted results of experiments. FP, HI-B, and DM drafted the manuscript. All authors edited, revised, and approved final version of manuscript.

### Conflict of interest statement

The authors declare that the research was conducted in the absence of any commercial or financial relationships that could be construed as a potential conflict of interest.

## References

[B1] Aiken S. J. Picton T. W. (2006). Envelope following responses to natural vowels. Audiol. Neurootol. 11, 213–232. 10.1159/000092589 16612051

[B2] Aiken S. J. Picton T. W. (2008). Envelope and spectral frequency-following responses to vowel sounds. Hear. Res. 245, 35–47. 10.1016/j.heares.2008.08.004 18765275

[B3] Audacity (2017). Audacity(R): Free Audio Editor and Recorder [Computer application]. Version *2.2.2*.

[B4] Bernstein J. G. Oxenham A. J. (2003). Pitch discrimination of diotic and dichotic tone complexes: harmonic resolvability or harmonic number? J. Acoust. Soc. Am. 113, 3323–3334. 10.1121/1.1572146 12822804

[B5] Bidelman G. M. (2018). Subcortical sources dominate the neuroelectric auditory frequency-following response to speech. Neuroimage 175, 56–69. 10.1016/j.neuroimage.2018.03.060 29604459

[B6] Boersma P. Weenink D. J. M. (2009). Praat: Doing Phonetics by Computer (Version 5.1.13).

[B7] Cariani P. A. Delgutte B. (1996a). Neural correlates of the pitch of complex tones. I. Pitch and pitch salience. J. Neurophysiol. 76, 1698–1716. 8890286 10.1152/jn.1996.76.3.1698

[B8] Cariani P. A. Delgutte B. (1996b). Neural correlates of the pitch of complex tones. II. Pitch shift, pitch ambiguity, phase invariance, pitch circularity, rate pitch, and the dominance region for pitch. J. Neurophysiol. 76, 1717–1734. 8890287 10.1152/jn.1996.76.3.1717

[B9] Chao Y. R. (1965). A Grammar of Spoken Chinese. Berkeley, CA: University of California Press.

[B10] Coffey E. B. Chepesiuk A. M. Herholz S. C. Baillet S. Zatorre R. J. (2017a). Neural correlates of early sound encoding and their relationship to speech-in-noise perception. Front. Neurosci. 11:479. 10.3389/fnins.2017.00479 28890684 PMC5575455

[B11] Coffey E. B. Colagrosso E. M. Lehmann A. Schönwiesner M. Zatorre R. J. (2016a). Individual differences in the frequency-following response: relation to pitch perception. PLoS ONE 11:e0152374. 10.1371/journal.pone.0152374 27015271 PMC4807774

[B12] Coffey E. B. Herholz S. C. Chepesiuk A. M. Baillet S. Zatorre R. J. (2016b). Cortical contributions to the auditory frequency-following response revealed by MEG. Nat. Commun. 7:11070. 10.1038/ncomms11070 27009409 PMC4820836

[B13] Coffey E. B. J. Musacchia G. Zatorre R. J. (2017b). Cortical correlates of the auditory frequency-following and onset responses: EEG and fMRI evidence. J. Neurosci. 37, 830–838. 10.1523/JNEUROSCI.1265-16.2016 28123019 PMC6597017

[B14] Dajani H. R. Purcell D. Wong W. Kunov H. Picton T. W. (2005). Recording human evoked potentials that follow the pitch contour of a natural vowel. IEEE Trans. Biomed. Eng. 52, 1614–1618. 10.1109/TBME.2005.851499 16189976

[B15] Fu Q.-J. Zeng F.-G. (2000). Identification of temporal envelope cues in Chinese tone recognition. Asia Pacific J. Speech Lang. Hear. 5, 45–57. 10.1179/136132800807547582

[B16] Fu Q.-J. Zeng F.-G. Shannon R. V. Soli S. D. (1998). Importance of tonal envelope cues in Chinese speech recognition. J. Acoust. Soc. Am. 104, 505–510. 10.1121/1.423251 9670541

[B17] Galbraith G. C. (1994). Two-channel brain-stem frequency-following responses to pure tone and missing fundamental stimuli. Electroencephalogr. Clin. Neurophysiol. Evok. Potent. Sect. 92, 321–330. 10.1016/0168-5597(94)90100-7 7517854

[B18] Glasberg B. R. Moore B. C. (1990). Derivation of auditory filter shapes from notched-noise data. Hear. Res. 47, 103–138. 10.1016/0378-5955(90)90170-T 2228789

[B19] Glaser E. Suter C. Dasheiff R. Goldberg A. (1976). The human frequency-following response: its behavior during continuous tone and tone burst stimulation. Clin. Neurophysiol. 40, 25–32. 10.1016/0013-4694(76)90176-0 55345

[B20] Gockel H. Moore B. C. Plack C. J. Carlyon R. P. (2006). Effect of noise on the detectability and fundamental frequency discrimination of complex tones. J. Acoust. Soc. Am. 120, 957–965. 10.1121/1.2211408 16938983

[B21] Gockel H. E. Carlyon R. P. Mehta A. Plack C. J. (2011). The frequency following response (FFR) may reflect pitch-bearing information but is not a direct representation of pitch. J. Assoc. Res. Otolaryngol. 12, 767–782. 10.1007/s10162-011-0284-1 21826534 PMC3214239

[B22] Gockel H. E. Krugliak A. Plack C. J. Carlyon R. P. (2015). Specificity of the human frequency following response for carrier and modulation frequency assessed using adaptation. J. Assoc. Res. Otolaryngol. 16, 747–762. 10.1007/s10162-015-0533-9 26162415 PMC4636589

[B23] Greenberg S. Marsh J. T. Brown W. S. Smith J. C. (1987). Neural temporal coding of low pitch. I. Human frequency-following responses to complex tones. Hear. Res. 25, 91–114. 10.1016/0378-5955(87)90083-9 3558136

[B24] Greenberg S. Smith J. Marsh J. Brown W. (1978). Human frequency following response to synthetic vowels. J. Acoust. Soc. Am. 63, S76–S76.

[B25] Houtsma A. J. M. Smurzynski J. (1990). Pitch identification and discrimination for complex tones with many harmonics. J. Acoust. Soc. Am. 87, 304–310. 10.1121/1.399297

[B26] Howie J. M. (1976). Acoustical Studies of Mandarin Vowels And Tones. New York, NY: Cambridge University Press.

[B27] Jeng F. C. Costilow C. E. Stangherlin D. P. Lin C. D. (2011). Relative power of harmonics in human frequency-following responses associated with voice pitch in American and Chinese adults. Percept. Mot. Skills 113, 67–86. 10.2466/10.24.PMS.113.4.67-86 21987910

[B28] Kaernbach C. Bering C. (2001). Exploring the temporal mechanism involved in the pitch of unresolved harmonics. J. Acoust. Soc. Am. 110, 1039–1048. 10.1121/1.1381535 11519572

[B29] Kong Y.-Y. Zeng F.-G. (2006). Temporal and spectral cues in Mandarin tone recognition. J. Acoust. Soc. Am. 120, 2830–2840. 10.1121/1.2346009 17139741

[B30] Krishnan A. (2002). Human frequency-following responses: representation of steady-state synthetic vowels. Hear. Res. 166, 192–201. 10.1016/S0378-5955(02)00327-1 12062771

[B31] Krishnan A. Bidelman G. M. Gandour J. T. (2010a). Neural representation of pitch salience in the human brainstem revealed by psychophysical and electrophysiological indices. Hear. Res. 268, 60–66. 10.1016/j.heares.2010.04.016 20457239 PMC3171186

[B32] Krishnan A. Bidelman G. M. Smalt C. J. Ananthakrishnan S. Gandour J. T. (2012). Relationship between brainstem, cortical and behavioral measures relevant to pitch salience in humans. Neuropsychologia 50, 2849–2859. 10.1016/j.neuropsychologia.2012.08.013 22940428 PMC3483071

[B33] Krishnan A. Gandour J. T. Bidelman G. M. (2010b). The effects of tone language experience on pitch processing in the brainstem. J. Neurolinguist. 23, 81–95. 10.1016/j.jneuroling.2009.09.001 20161561 PMC2805250

[B34] Krishnan A. Gandour J. T. Bidelman G. M. Swaminathan J. (2009). Experience dependent neural representation of dynamic pitch in the brainstem. Neuroreport 20, 408–413. 10.1097/WNR.0b013e3283263000 19223793 PMC2692950

[B35] Krishnan A. Plack C. J. (2011). Neural encoding in the human brainstem relevant to the pitch of complex tones. Hear. Res. 275, 110–119. 10.1016/j.heares.2010.12.008 21167923

[B36] Krishnan A. Xu Y. Gandour J. Cariani P. (2005). Encoding of pitch in the human brainstem is sensitive to language experience. Brain Res. Cogn. Brain Res. 25, 161–168. 10.1016/j.cogbrainres.2005.05.004 15935624

[B37] Krishnan A. Xu Y. Gandour J. T. Cariani P. A. (2004). Human frequency-following response: representation of pitch contours in Chinese tones. Hear. Res. 189, 1–12. 10.1016/S0378-5955(03)00402-7 14987747

[B38] Laroche M. Dajani H. R. Prévost F. Marcoux A. M. (2013). Brainstem auditory responses to resolved and unresolved harmonics of a synthetic vowel in quiet and noise. Ear Hear. 34, 63–74. 10.1097/AUD.0b013e31826119a1 22814487

[B39] Levitt H. (1971). Transformed up-down methods in psychoacoustics. J. Acoust. Soc. Am. 49, 467–477. 10.1121/1.1912375 5541744

[B40] Liu C. Azimi B. Bhandary M. Hu Y. (2014). Contribution of low-frequency harmonics to Mandarin Chinese tone identification in quiet and six-talker babble background. J. Acoust. Soc. Am. 135, 428–438. 10.1121/1.4837255 24437783

[B41] Luo X. Fu Q.-J. (2006). Contribution of low-frequency acoustic information to Chinese speech recognition in cochlear implant simulations. J. Acoust. Soc. Am. 120, 2260–2266. 10.1121/1.2336990 17069321

[B42] Marmel F. Linley D. Carlyon R. Gockel H. Hopkins K. Plack C. (2013). Subcortical neural synchrony and absolute thresholds predict frequency discrimination independently. J. Assoc. Res. Otolaryngol. 14, 757–766. 10.1007/s10162-013-0402-3 23760984 PMC3767871

[B43] Marsh J. T. Brown W. S. Smith J. C. (1975). Far-field recorded frequency-following responses: correlates of low pitch auditory perception in humans Responses d'entertainement enregistrees a distance: correlates de la perception auditive des sons de faible hauteur chez l'homme. Electroencephalogr. Clin. Neurophysiol. 38, 113–119. 10.1016/0013-4694(75)90220-5 45941

[B44] Meddis R. Hewitt M. J. (1991). Virtual pitch and phase sensitivity of a computer model of the auditory periphery. I: pitch identification. J. Acoust. Soc. Am. 89, 2866–2882. 10.1121/1.400725

[B45] Meddis R. O'mard L. (1997). A unitary model of pitch perception. J. Acoust. Soc. Am. 102, 1811–1820. 9301058 10.1121/1.420088

[B46] Moore B. C. (2012). An Introduction to the Psychology of Hearing. Boston, MA: Brill.

[B47] Mormann F. Lehnertz K. David P. Elger C. E. (2000). Mean phase coherence as a measure for phase synchronization and its application to the EEG of epilepsy patients. Physica D Nonlin. Phenom. 144, 358–369. 10.1016/S0167-2789(00)00087-7

[B48] Plack C. J. Oxenham A. J. Fay R. R. (2006). Pitch: Neural Coding and Perception. New York, NY: Springer Science & Business Media.

[B49] Plomp R. (1967). Pitch of complex tones. J. Acoust. Soc. Am. 41, 1526–1533. 10.1121/1.1910515 6075560

[B50] Russo N. Nicol T. Musacchia G. Kraus N. (2004). Brainstem responses to speech syllables. Clin. Neurophysiol. 115, 2021–2030. 10.1016/j.clinph.2004.04.003 15294204 PMC2529166

[B51] Shinn-Cunningham B. Varghese L. Wang L. Bharadwaj H. (2017). Individual differences in temporal perception and their implications for everyday listening, in The Frequency-Following Response, ed Shinn-Cunningham B. (Barbara, CA: Springer), 159–192.

[B52] Skoe E. Kraus N. (2010). Auditory brain stem response to complex sounds: a tutorial. Ear Hear. 31, 302–324. 10.1097/AUD.0b013e3181cdb272 20084007 PMC2868335

[B53] Smalt C. J. Krishnan A. Bidelman G. M. Ananthakrishnan S. Gandour J. T. (2012). Distortion products and their influence on representation of pitch-relevant information in the human brainstem for unresolved harmonic complex tones. Hear. Res. 292, 26–34. 10.1016/j.heares.2012.08.001 22910032 PMC3483078

[B54] Smith J. C. Marsh J. T. Brown W. S. (1975). Far-field recorded frequency-following responses: evidence for the locus of brainstem sources. Clin. Neurophysiol. 39, 465–472. 10.1016/0013-4694(75)90047-4 52439

[B55] Smith J. C. Marsh J. T. Greenberg S. Brown W. S. (1978). Human auditory frequency-following responses to a missing fundamental. Science 201, 639–641. 10.1126/science.675250 675250

[B56] Sohmer H. Pratt H. Kinarti R. (1977). Sources of frequency following responses (FFR) in man. Electroencephalogr. Clin. Neurophysiol. 42, 656–664. 10.1016/0013-4694(77)90282-6 67025

[B57] Song J. H. Skoe E. Wong P. C. Kraus N. (2008). Plasticity in the adult human auditory brainstem following short-term linguistic training. J. Cogn. Neurosci. 20, 1892–1902. 10.1162/jocn.2008.20131 18370594 PMC2829864

[B58] Stagray J. R. Downs D. Sommers R. K. (1992). Contributions of the fundamental, resolved harmonics, and unresolved harmonics in tone-phoneme identification. J. Speech Lang. Hear. Res. 35, 1406–1409. 10.1044/jshr.3506.1406 1494283

[B59] Tallon-Baudry C. Bertrand O. Delpuech C. Pernier J. (1996). Stimulus specificity of phase-locked and non-phase-locked 40 Hz visual responses in human. J. Neurosci. 16, 4240–4249. 10.1523/JNEUROSCI.16-13-04240.1996 8753885 PMC6579008

[B60] Whalen D. H. Xu Y. (1992). Information for Mandarin tones in the amplitude contour and in brief segments. Phonetica 49, 25–47. 10.1159/000261901 1603839

[B61] Worden F. G. Marsh J. T. (1968). Frequency-following (microphonic-like) neural responses evoked by sound. Electroencephalogr. Clin. Neurophysiol. 25, 42–52. 10.1016/0013-4694(68)90085-0 4174782

[B62] Xu L. Pfingst B. E. (2003). Relative importance of temporal envelope and fine structure in lexical-tone perception (L). J. Acoust. Soc. Am. 114, 3024–3027. 10.1121/1.1623786 14714781 PMC1283139

[B63] Zhu L. Bharadwaj H. Xia J. Shinn-Cunningham B. (2013). A comparison of spectral magnitude and phase-locking value analyses of the frequency-following response to complex tones. J. Acoust. Soc. Am. 134, 384–395. 10.1121/1.4807498 23862815 PMC3724813

